# Supplementary material for: Domino-like multi-emissions across red and near infrared from solid-state 2-/2,6-aryl substituted BODIPY dyes
Source: Nat Commun. 2018 Jul 12;9:2688. doi: 10.1038/s41467-018-05040-8 (PMC6043560; doi:10.1038/s41467-018-05040-8)
Supplement: Supplementary file 1 — Supplementary Information [file 41467_2018_5040_MOESM1_ESM.pdf]

**Domino-Like Multi-Emissions across Red and Near Infra-Red from Solid-State 2-/2,6-Aryl Substituted BODIPY Dyes**

Tian, *et al*.

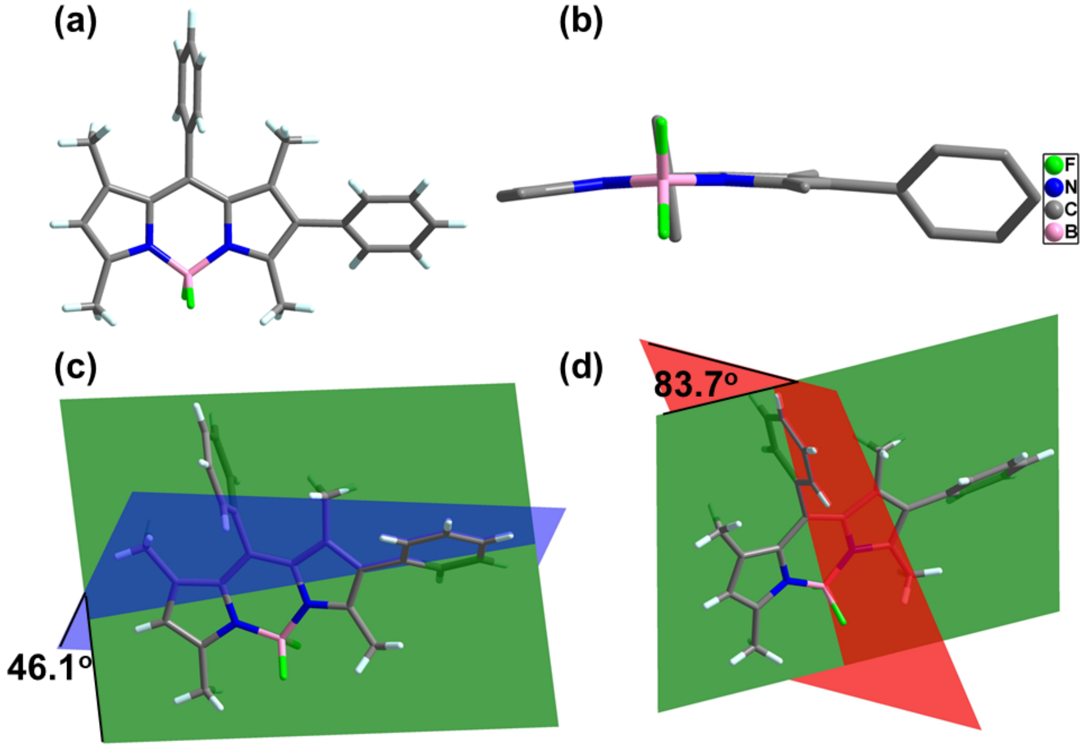


**Supplementary Figure 1.** (a) Top and (b) side view of single crystal X-ray diffraction structure of **BDP1**. The dihedral angles between (c) 2- and (d) 8-phenyl substituent and indacene plane.

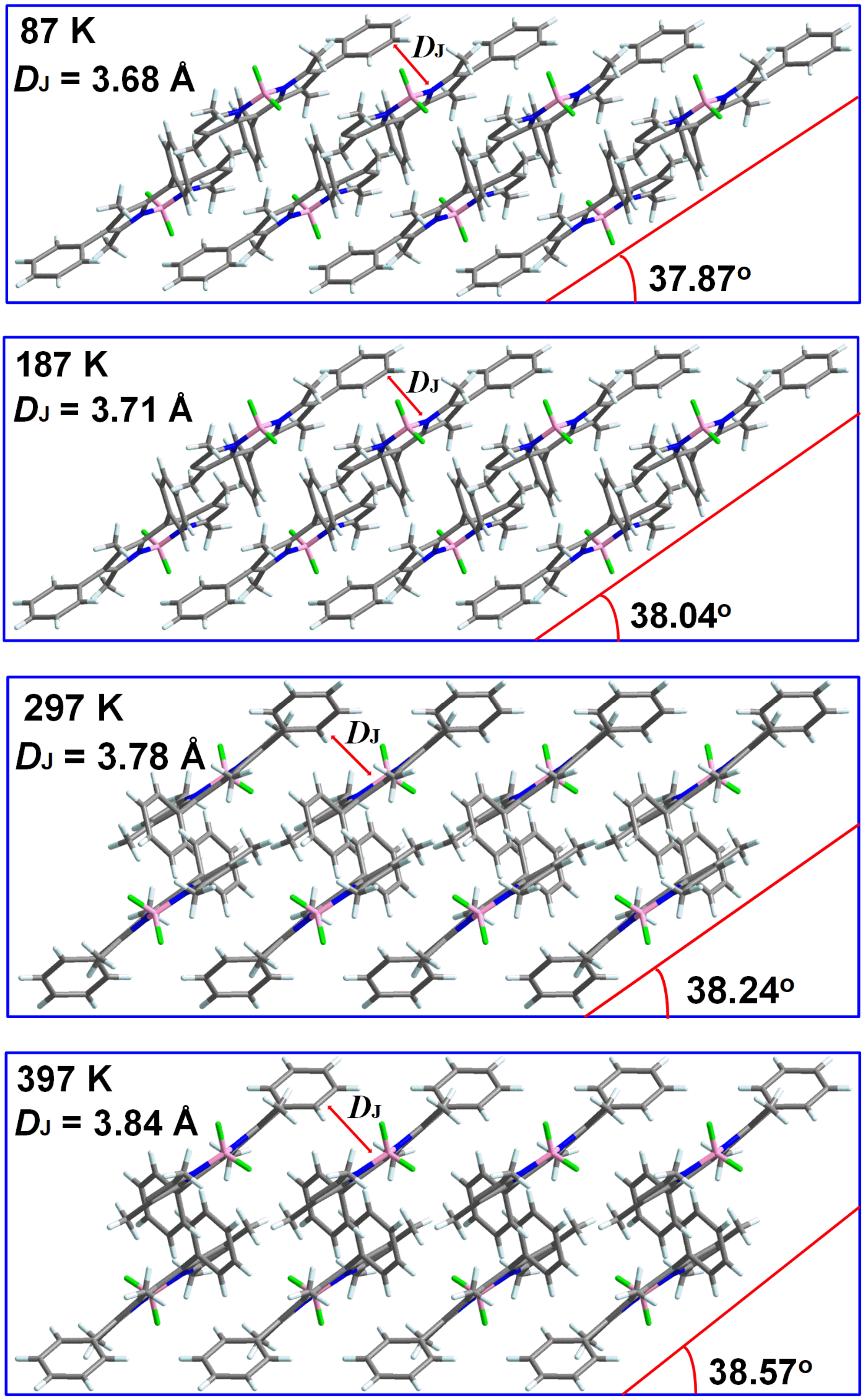


**Supplementary Figure 2.** Variations of the molecular packing mode at different temperatures viewed along the *c*-axis in the single crystal of **BDP1**.

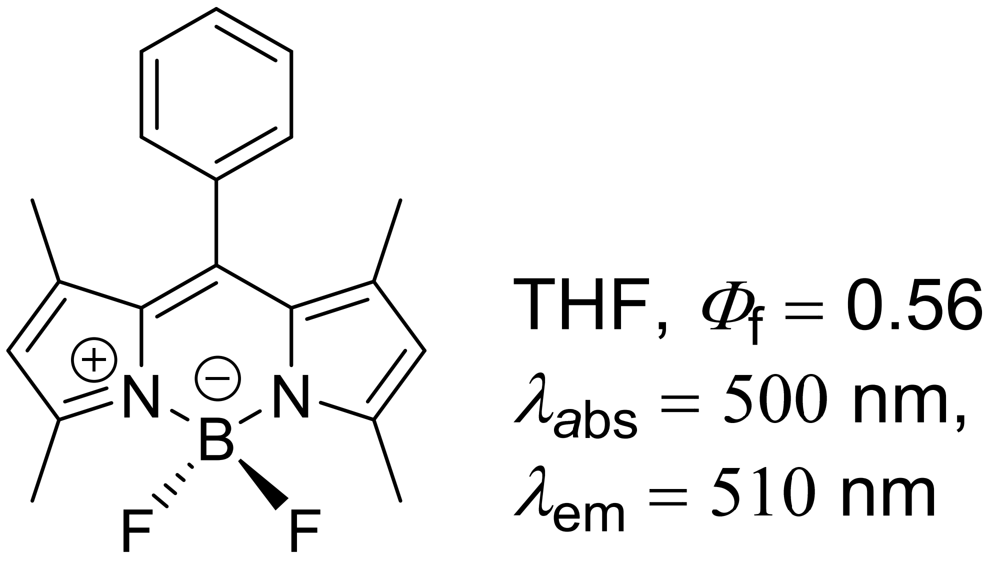


**BDP**

**Supplementary Figure 3.** Molecular structure and photophysical properties of **BDP**.

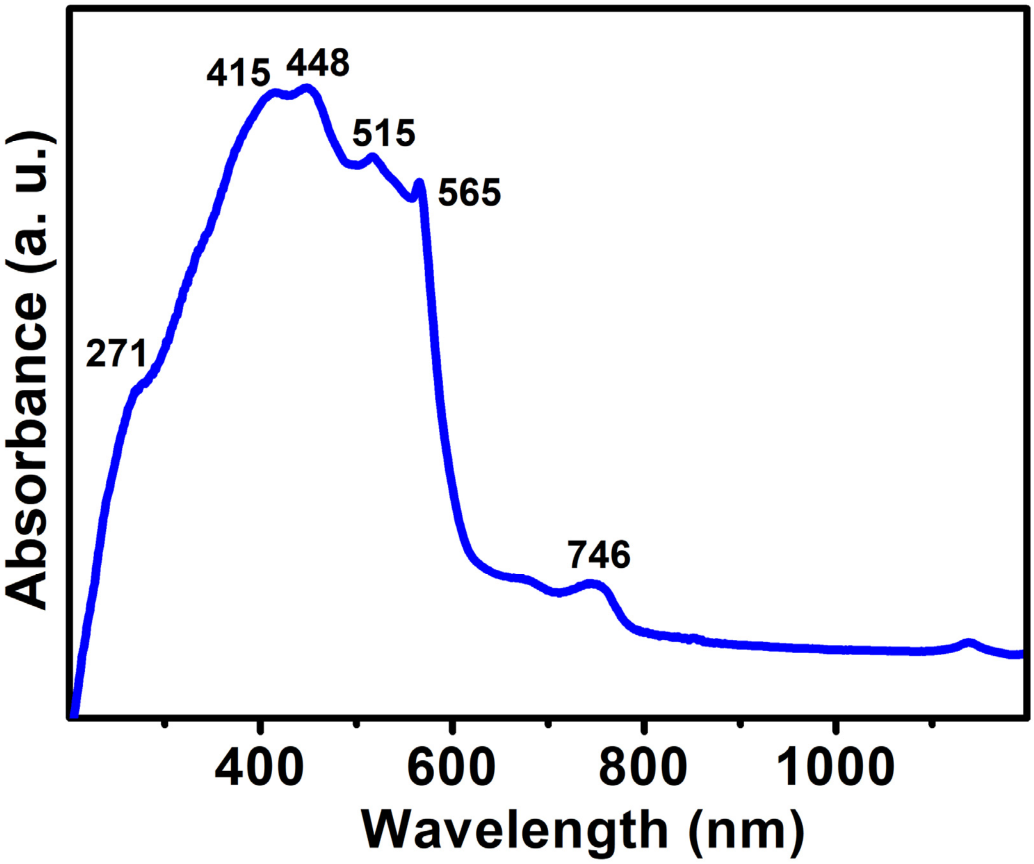


**Supplementary Figure 4.** UV-vis absorption spectrum of microcrystalline powder state **BDP1**.

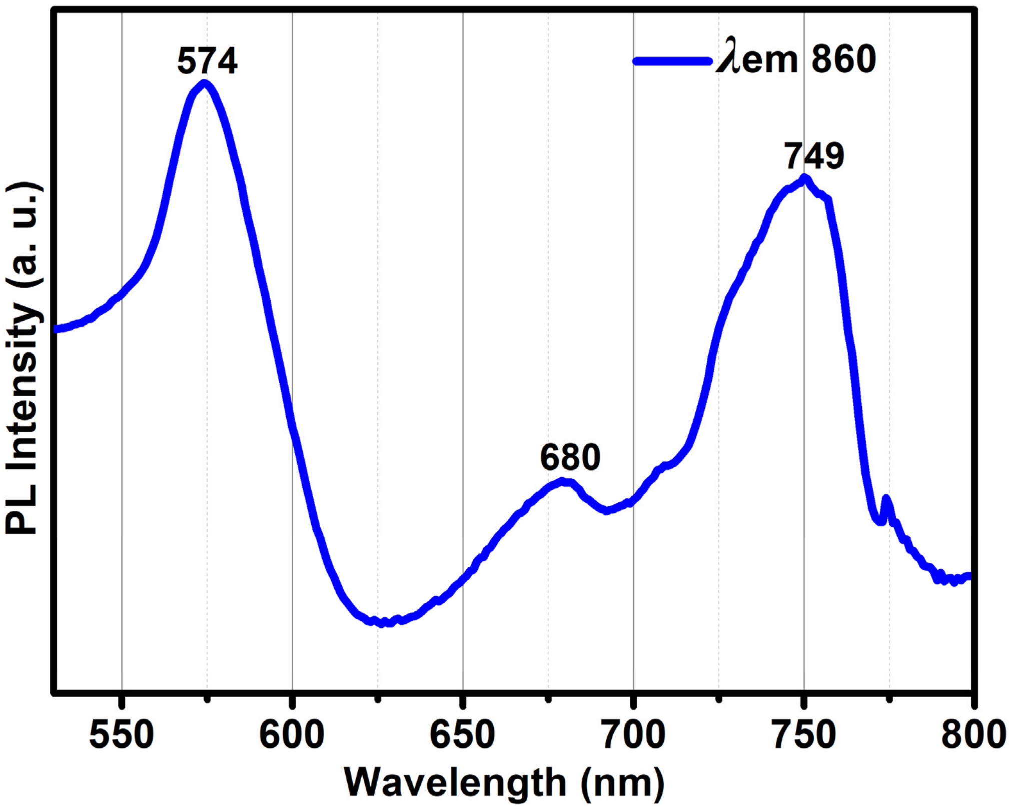


**Supplementary Figure 5.** Excitation spectrum of microcrystalline powder state **BDP1** when the emission was fixed at 860 nm.

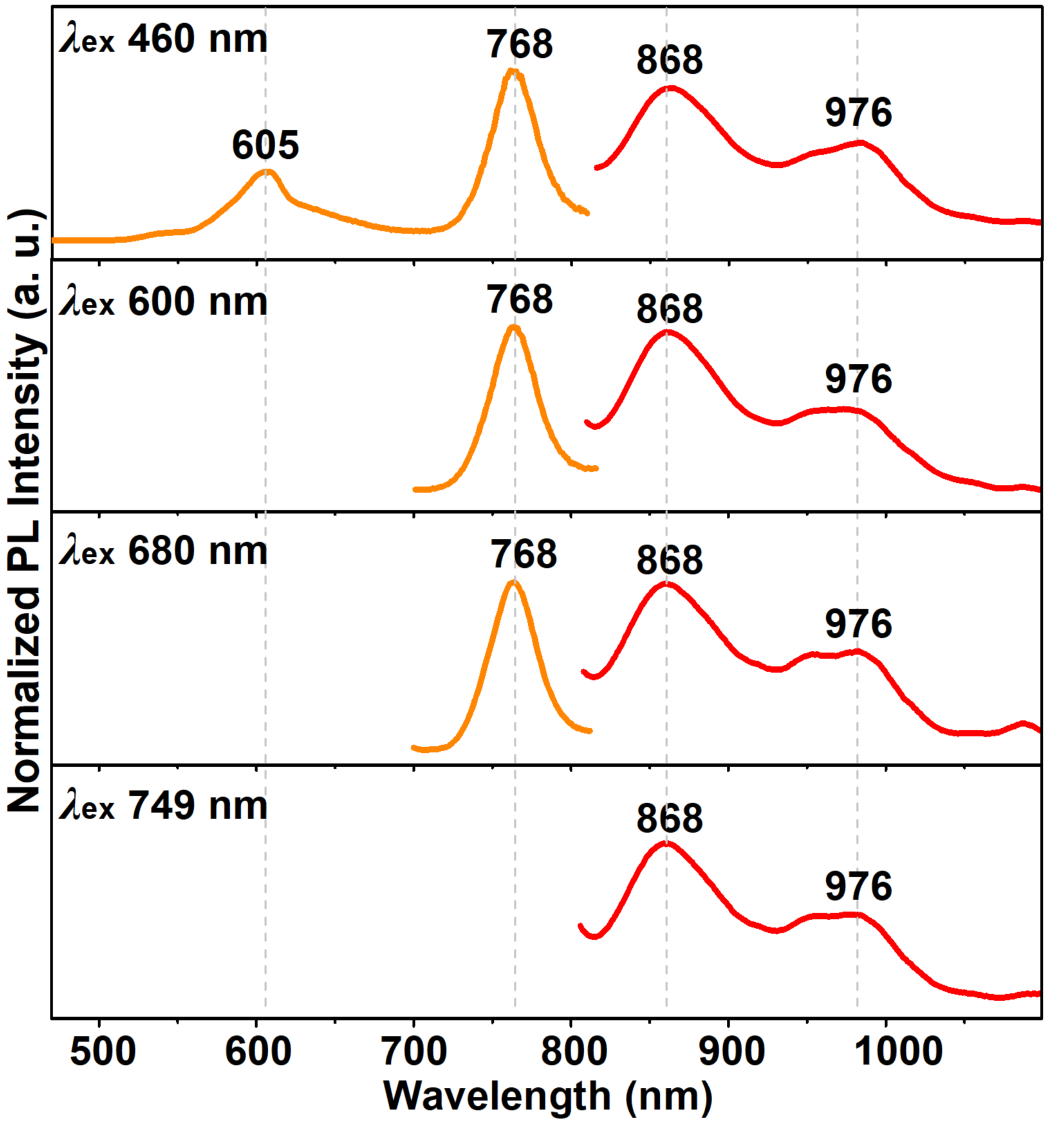


**Supplementary Figure 6.** Fluorescence spectra of microcrystalline powder state **BDP1** excited with light at different wavelengths (460, 600, 680, and 749 nm).

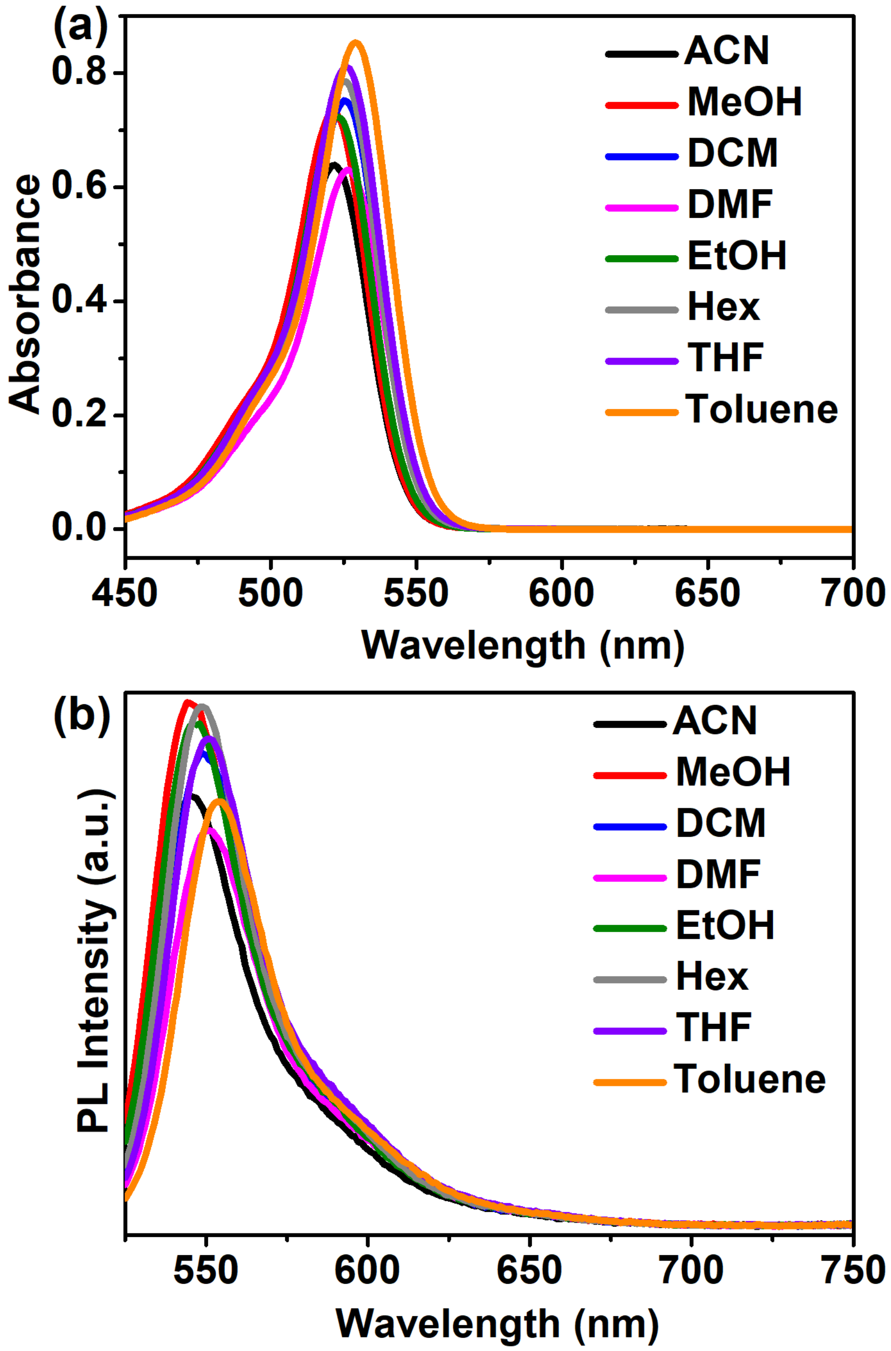


**Supplementary Figure 7.** Absorption and emission spectra (slit width: 1 nm, 0.5 nm) of **BDP1** ($1 \times 10^{-5}$ mol $L^{-1}$) in different solvents (ACN: Acetonitrile; MeOH: Methanol; DCM: Dichloromethane; DMF: Dimethylforamide; EtOH: Ethanol; Hex: Hexane; THF: Tetrahydrofuran).

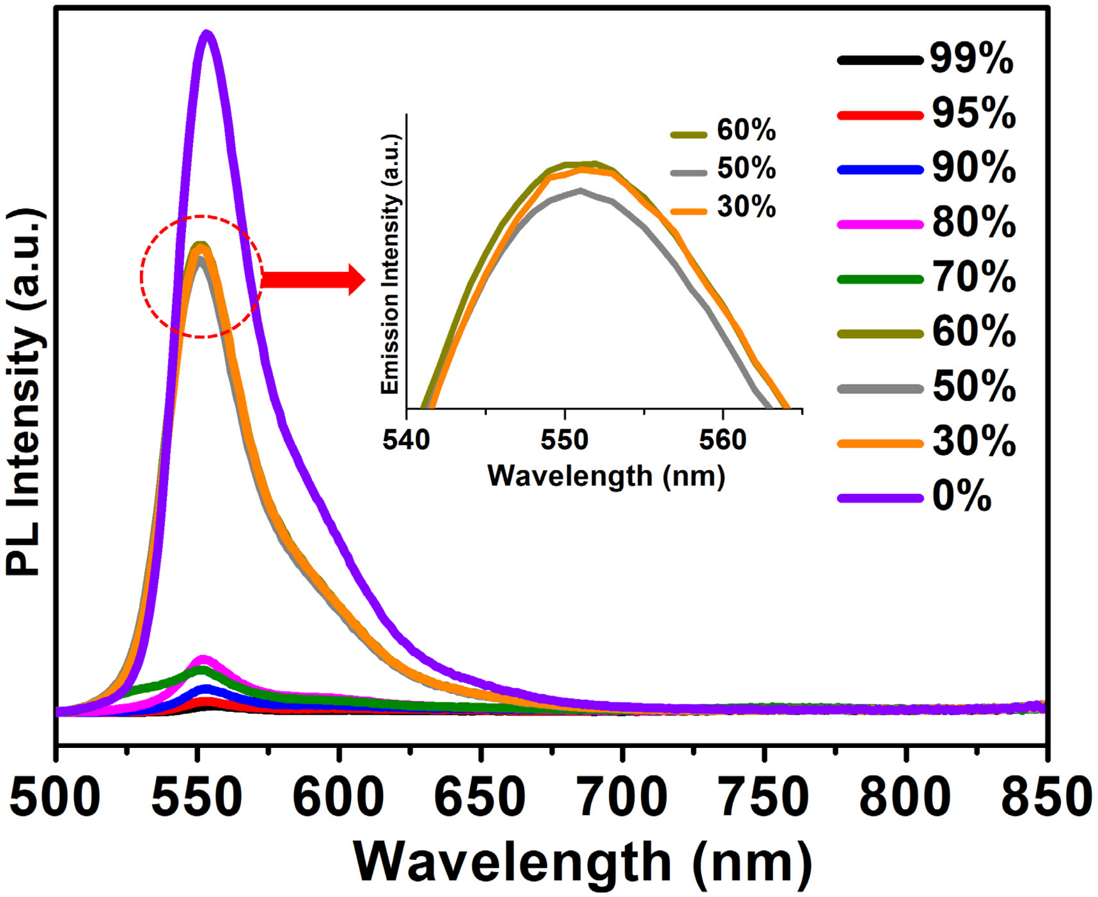


**Supplementary Figure 8.** Fluorescence spectra of **BDP1** ($1 \times 10^{-5}$ mol $L^{-1}$) in THF solution mixed with varied volumetric fractions of water ($f_w$) ($\lambda_{ex}$ = 480 nm, slit width: 1 nm, 1 nm).

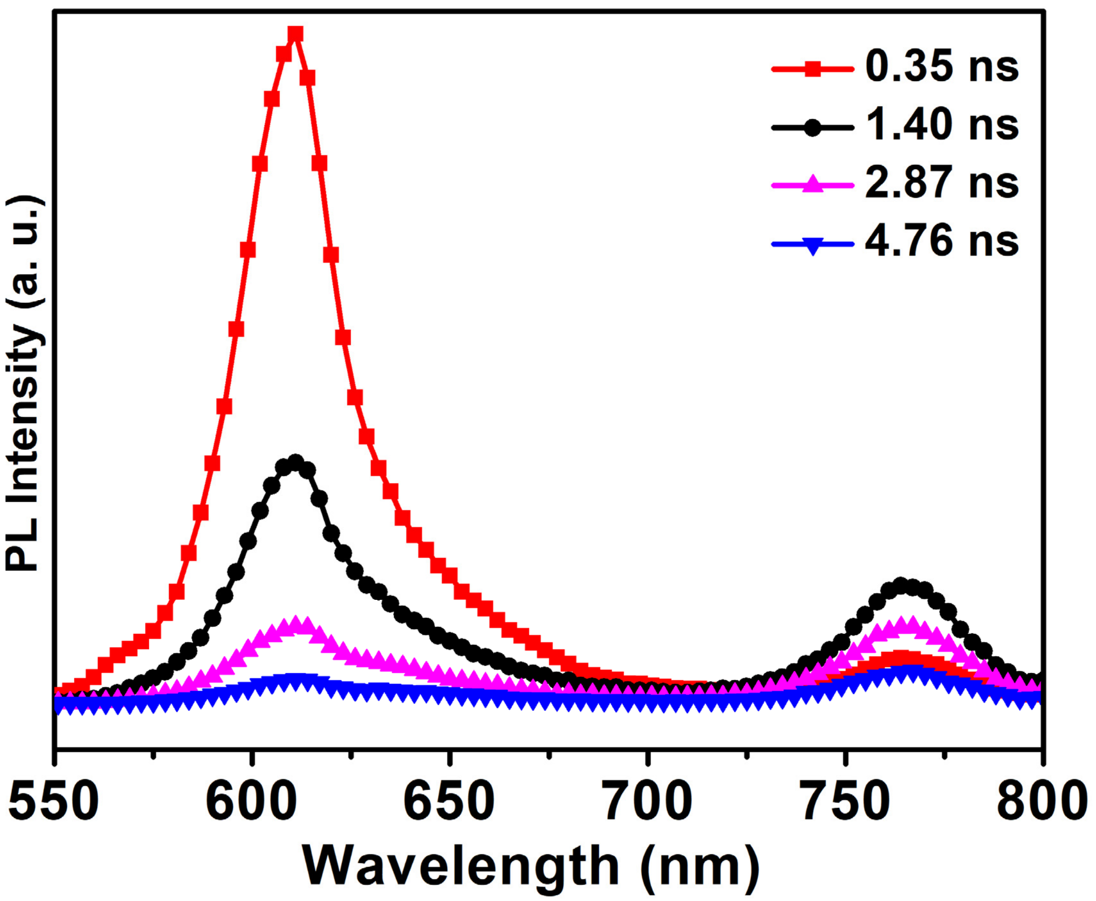


**Supplementary Figure 9.** Time-resolved fluorescence spectra of microcrystalline powder state **BDP1** at 0.35, 1.40, 2.87, and 4.76 ns.

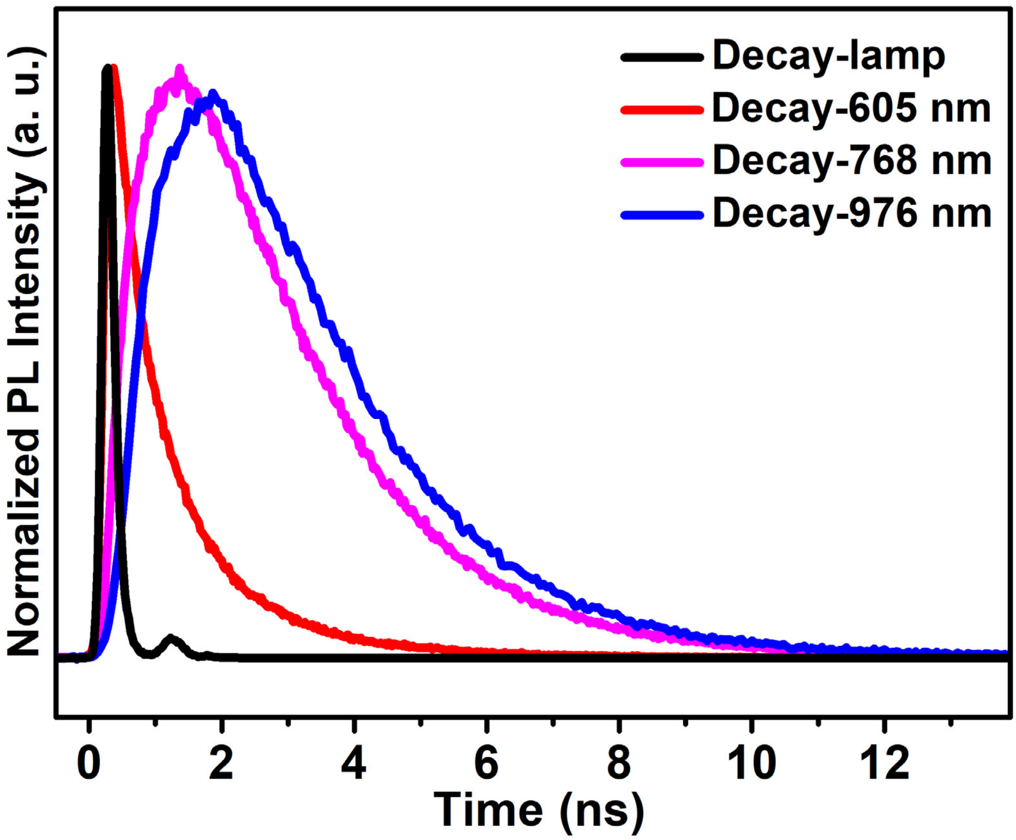


**Supplementary Figure 10.** Fluorescence decay curves of **BDP1** at 605, 768, and 976 nm. All samples measured were in the microcrystalline powder state.

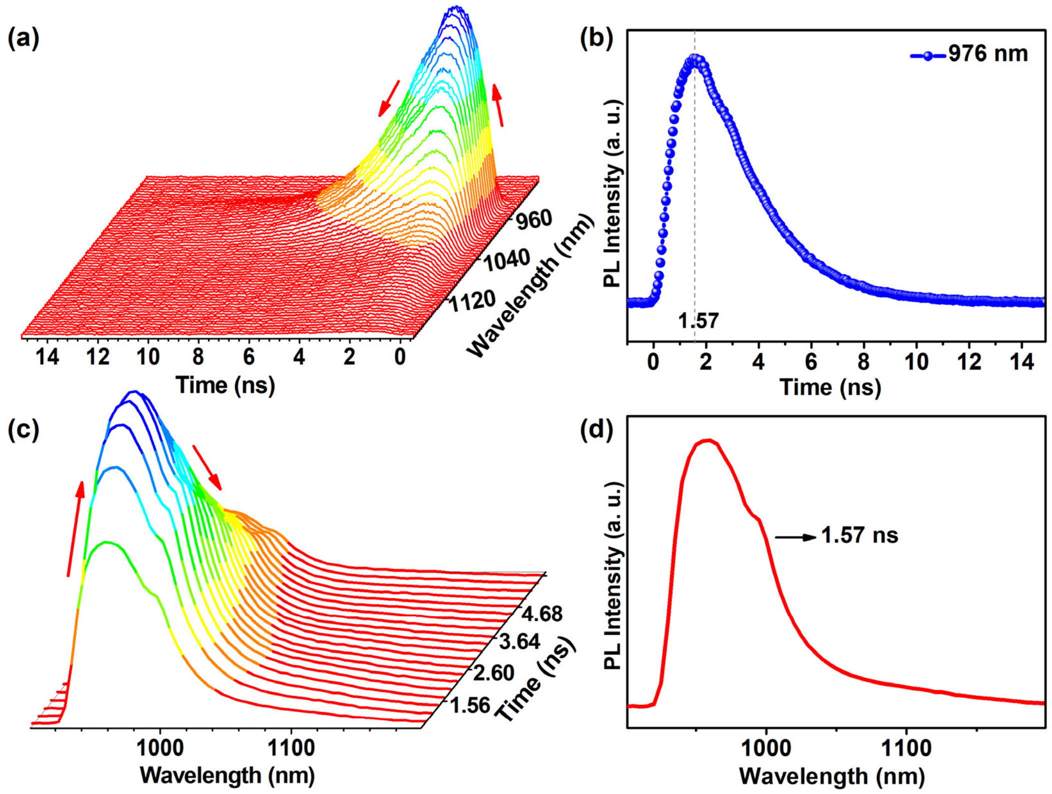


**Supplementary Figure 11.** (a) Fluorescence decay curves of **BDP1**. The emission wavelength ($\lambda_{em}$) varied from 900 to 1200 nm. (b) Fluorescence decay curve of **BDP1** at emission wavelength of 976 nm. (c) Time-resolved emission spectra of **BDP1**. (d) Time-resolved fluorescence spectra of **BDP1** at 1.57 ns. All samples measured were in the microcrystalline powder state.

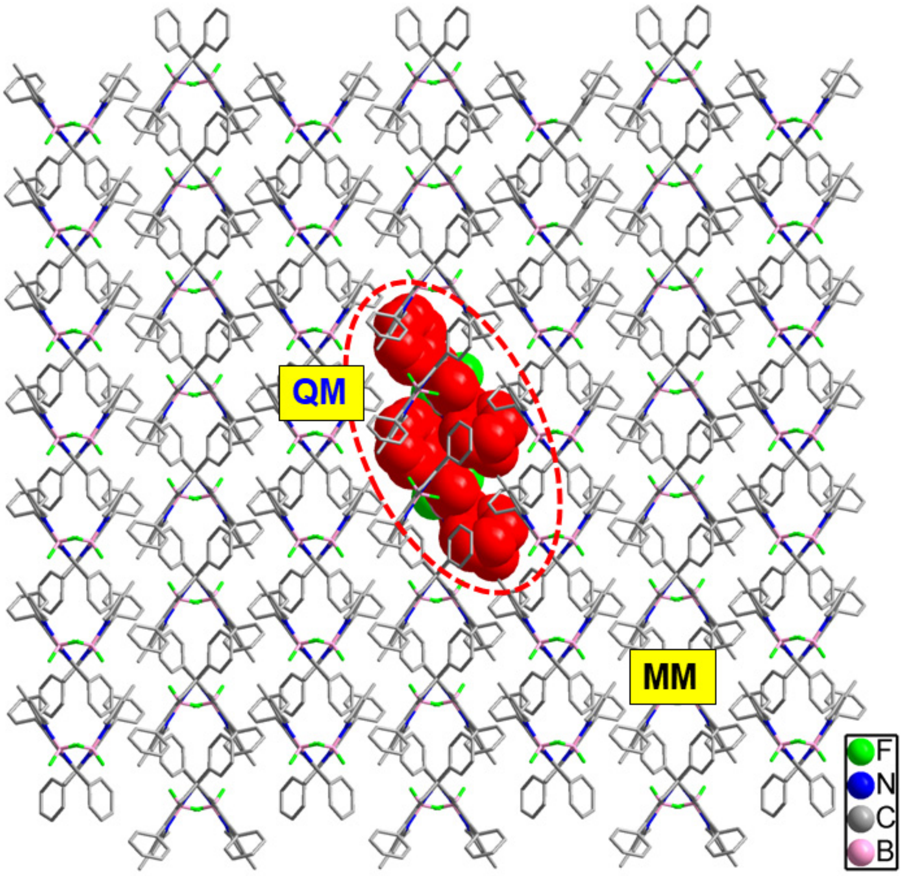


**Supplementary Figure 12.** Set up of QM/MM model for **BPD1** taking QM dimer as an example.

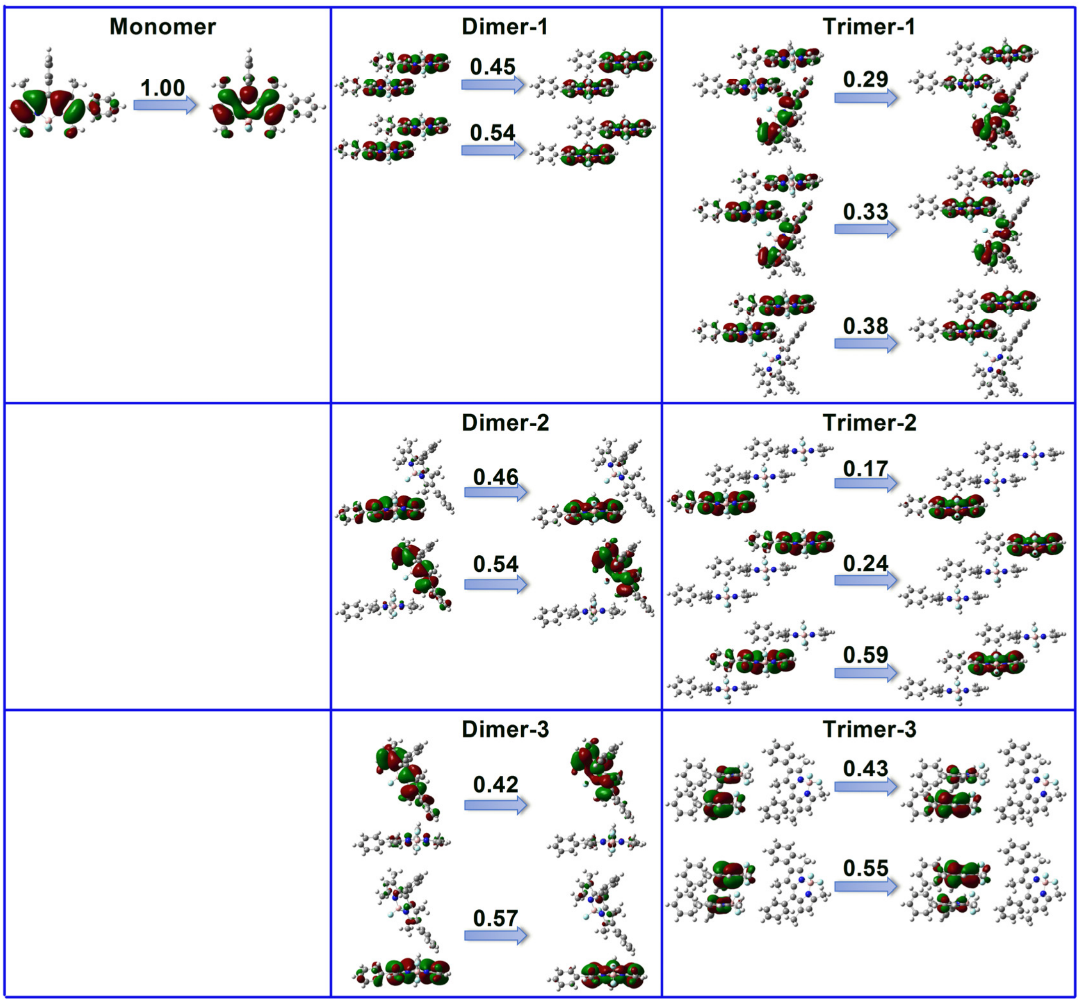


**Supplementary Figure 13.** Calculated NTOs of **BDP1** monomer, dimer-1, and trimer-1 for the lowest singlet states ($S_1$).

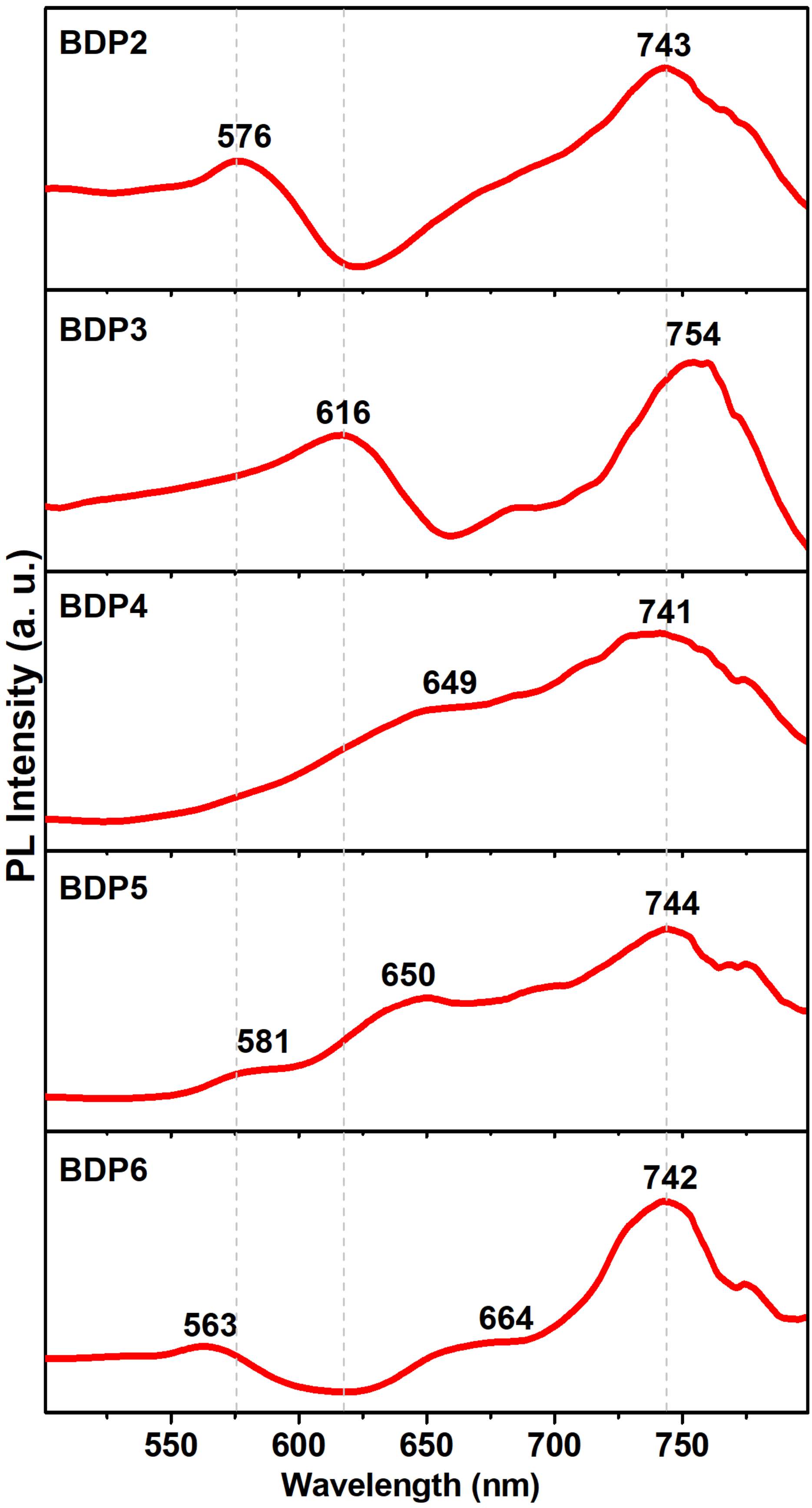


**Supplementary Figure 14.** Excitation spectra of **BDP2-6** in microcrystalline powder state when the emission was fixed at 900 nm.

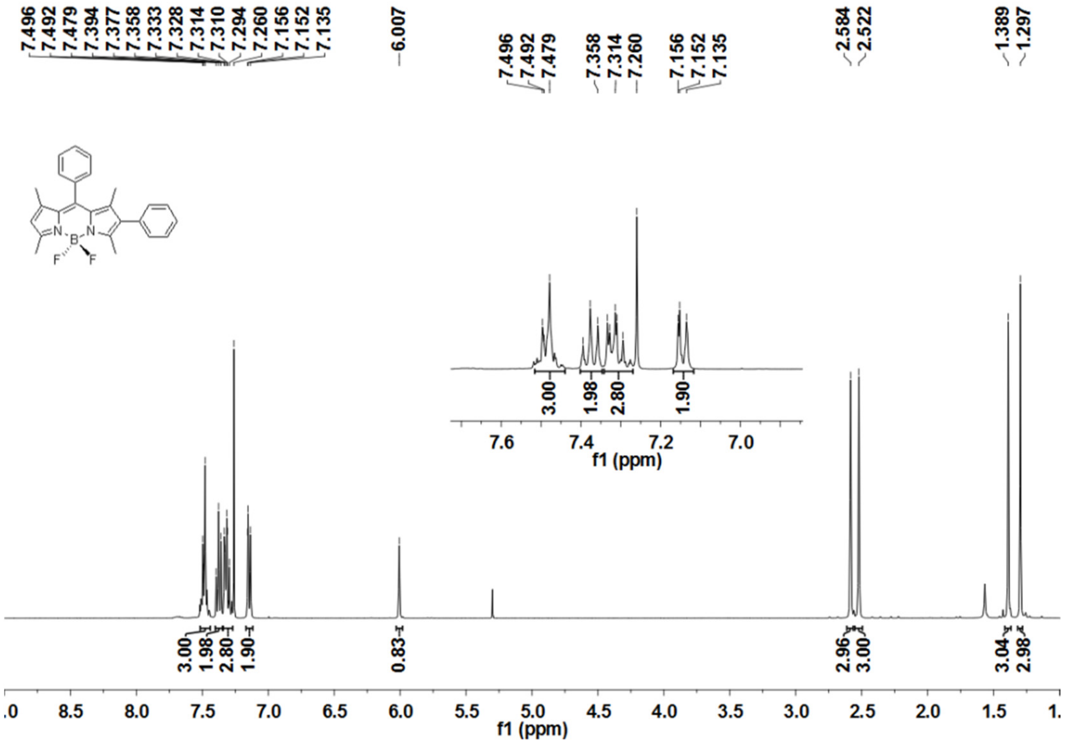

**Supplementary Figure 15.** $^{1}H$ NMR of **BDP1** in $CDCl_3$.

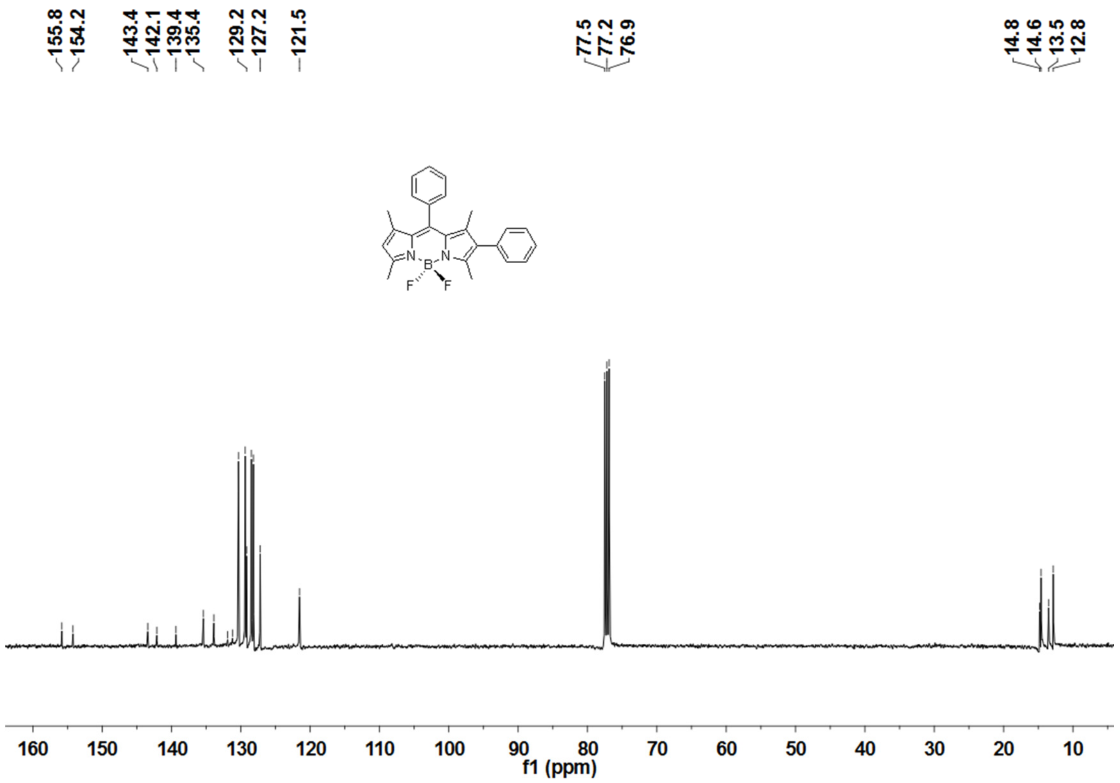


**Supplementary Figure 16.** $^{13}$C NMR of **BDP1** in $CDCl_3$.

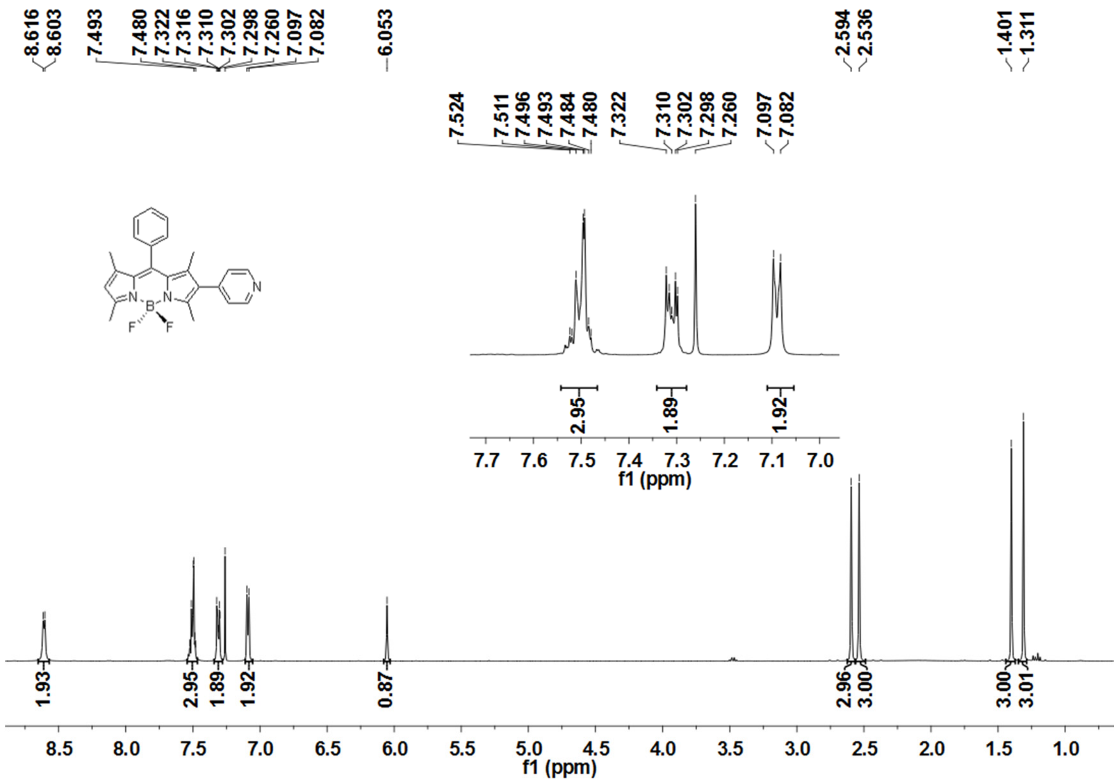


**Supplementary Figure 17.** $^1$H NMR of **BDP4** in $CDCl_3$.

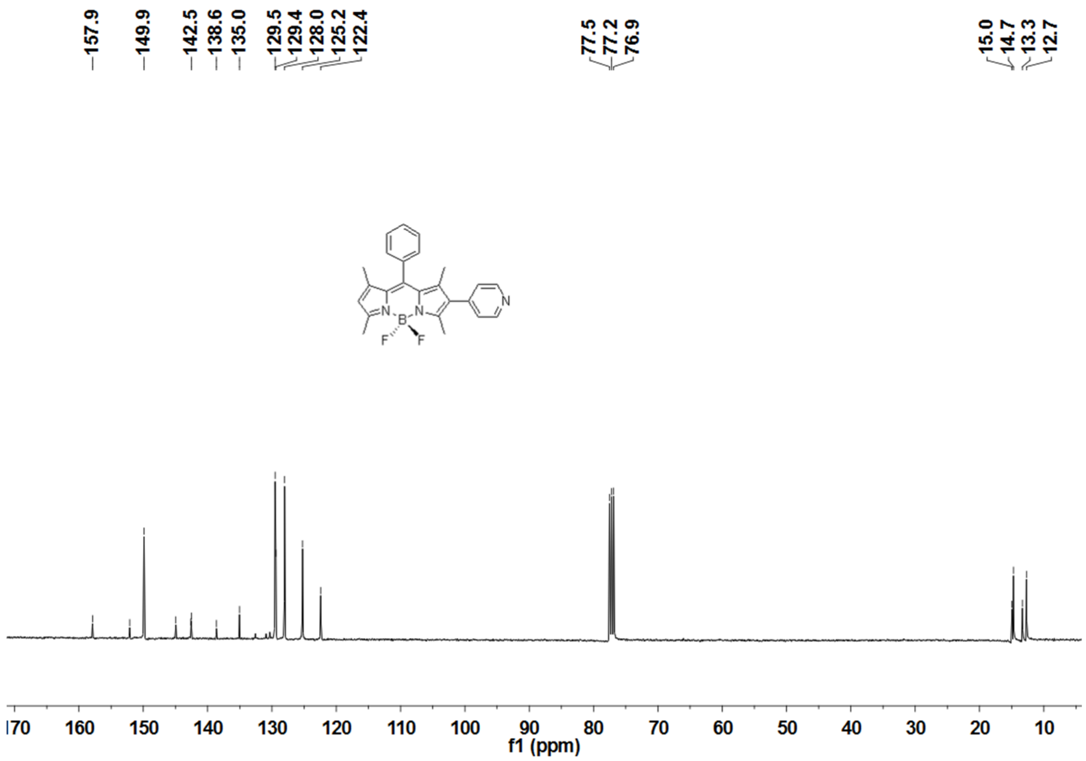


**Supplementary Figure 18.** $^{13}$C NMR of **BDP4** in $CDCl_3$.

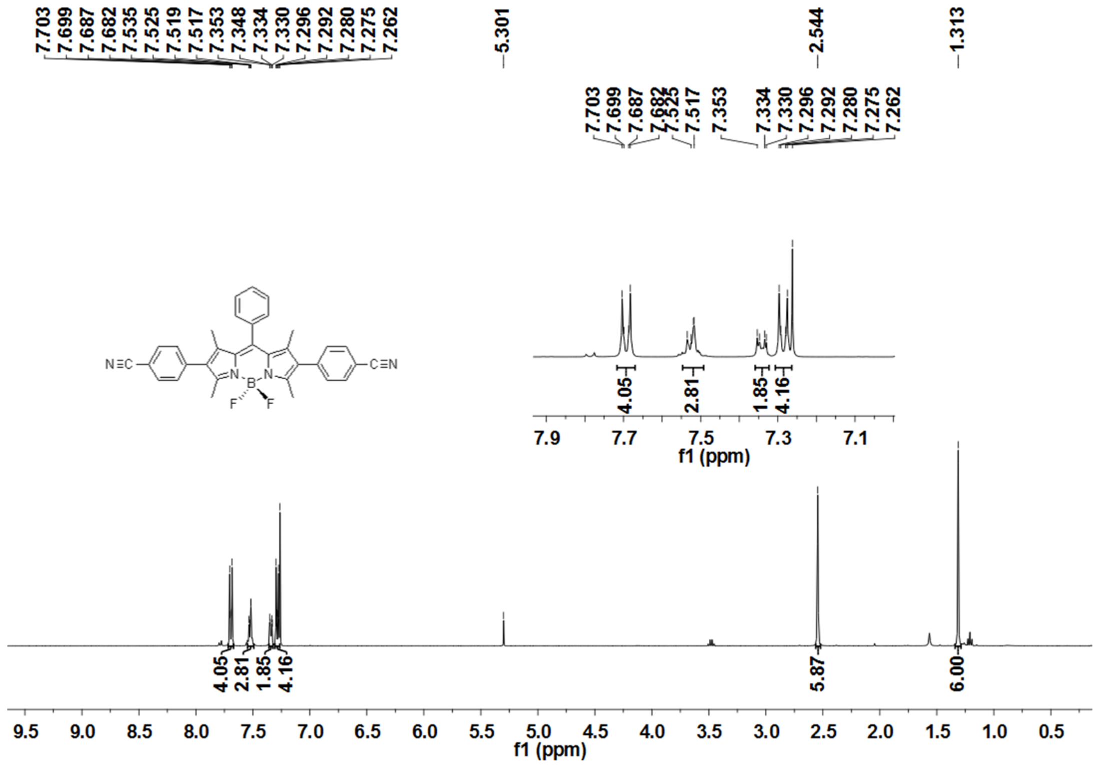


**Supplementary Figure 19.** $^{1}$H NMR of **BDP6** in $CDCl_3$.

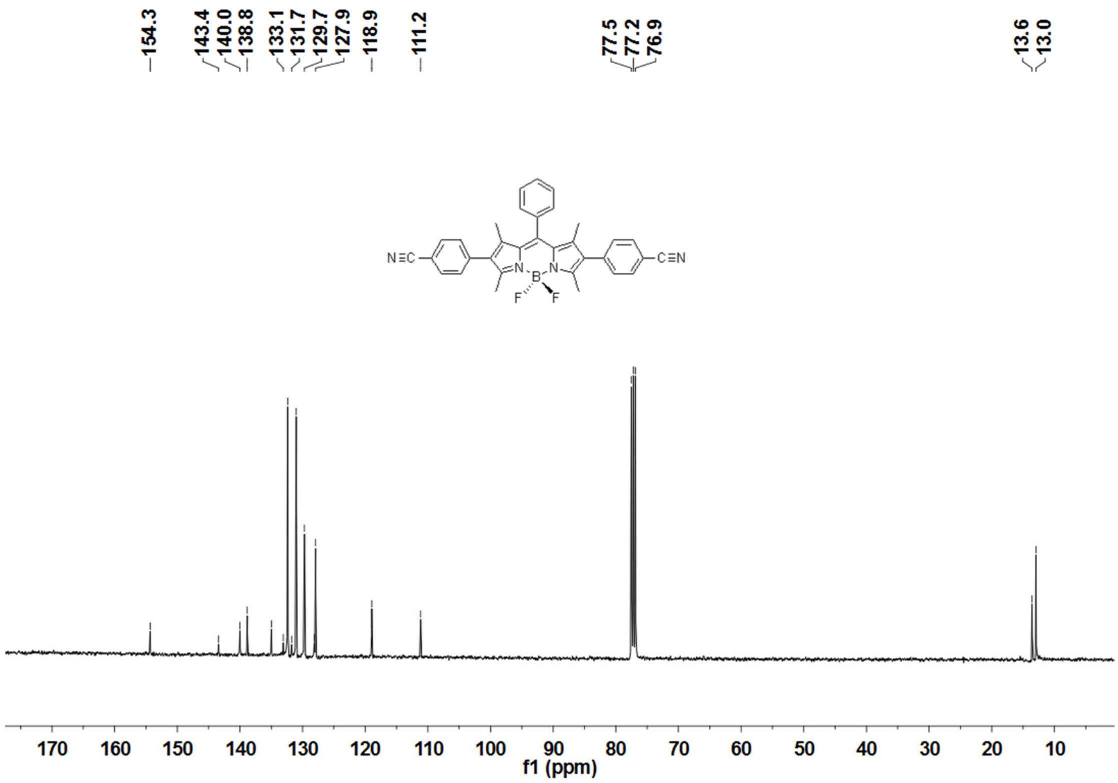


**Supplementary Figure 20.** $^{13}$C NMR of **BDP6** in $CDCl_3$.

**Supplementary Table 1** Packing parameters of single crystal **BDP1** at different temperatures

| | $\theta_J$ (°) | $a$ (Å) | $b$ (Å) | $c$ (Å) | $\alpha$ (°) | $\beta$ (°) | $\gamma$ (°) | $V$ (Å$^3$) |
|---|---|---|---|---|---|---|---|---|
| **87 K** | 37.87 | 6.9797 | 15.278 | 18.956 | 90 | 94.895 | 90 | 2014.20 |
| **187 K** | 38.04 | 6.9985 | 15.496 | 18.927 | 90 | 94.112 | 90 | 2045.19 |
| **297 K** | 38.24 | 7.0285 | 15.740 | 19.565 | 90 | 106.019 | 90 | 2080.40 |
| **397 K** | 38.57 | 7.0667 | 15.950 | 19.556 | 90 | 105.993 | 90 | 2118.92 |

**Supplementary Table 2** Fluorescence lifetimes of microcrystalline powder state **BDP1**

| **$\lambda_{em}$ (nm)** | **605** | **768** | **976** |
|---|---|---|---|
| $\tau_1$ (ns) | 0.83 | 0.73 | 0.99 |
| $a_1$ (%) | 55.61 | -62.79 | -91.86 |
| $\tau_2$ (ns) | 2.89 | 1.37 | 12.9 |
| $a_2$ (%) | 5.96 | 5.53 | 3.65 |
| $\tau_3$ (ns) | 1.88 | 1.93 | 1.84 |
| $a_3$ (%) | 38.43 | 157.27 | 188.22 |
| Average Lifetime | 0.92 | 1.31 | 1.46 |

**Supplementary Table 3** Calculated vertical excitation energies and oscillator strength (*f*) of the lowest singlet states ($S_1$) for monomer, dimer, and trimer of **BDP1**

| **$S_1$** | **monomer** | **Dimer** | | | **Trimer** | | |
|---|---|---|---|---|---|---|---|
| | | **1** | **2** | **3** | **1** | **2** | **3** |
| ***E* (eV)** | 2.960 | 2.951 | 2.947 | 2.962 | 2.912 | 2.916 | 2.946 |
| ***f*** | 0.647 | 1.263 | 0.345 | 0.346 | 0.654 | 1.786 | 1.026 |

**Supplementary Table 4** Crystallographic data and structure refinement for **BDP1**

| | **BDP1-87 K** | **BDP1-187 K** | **BDP1-297 K** | **BDP1-397 K** |
|---|---|---|---|---|
| CCDC number | **1540363** | **1837300** | **1540364** | **1837301** |
| Formula | $C_{25}H_{23}BF_2N_2$ | $C_{25}H_{23}BF_2N_2$ | $C_{25}H_{23}BF_2N_2$ | $C_{25}H_{23}BF_2N_2$ |
| $M_w$ | 400.26 | 400.26 | 400.26 | 400.26 |
| Temperature (K) | 87 K | 187 K | 297 K | 397 K |
| Wavelength (Å) | Mo K$\alpha$ | Mo K$\alpha$ | Mo K$\alpha$ | Mo K$\alpha$ |
| | ($\lambda$ = 0.71073) | ($\lambda$ = 0.71073) | ($\lambda$ = 0.71073) | ($\lambda$ = 0.71073) |
| Crystal system | monoclinic | monoclinic | monoclinic | monoclinic |
| Space group | $P2_1$/n | $P2_1$/n | $P2_1$/c | $P2_1$/c |
| Cell dimensions | $a$ = 6.9797(4)Å | $a$ = 6.9985(4) Å | $a$ = 7.0285(16) Å | $a$ = 7.0667(14) Å |
| | $b$ = 15.278(8) Å | $b$ = 15.4964(9) Å | $b$ = 15.74(4) Å | $b$ = 15.95(4) Å |
| | $c$ = 18.956(11) Å | $c$ =18.9276(13) Å | $c$ = 19.565(4) Å | $c$ = 19.556(4) Å |
| | $\alpha$ = 90° | $\alpha$ = 90° | $\alpha$ = 90° | $\alpha$ = 90° |
| | $\beta$ = 94.895(2)° | $\beta$ = 94.911(2) ° | $\beta$ = 106.019(9) ° | $\beta$ = 105.993(8) ° |
| | $\gamma$ = 90° | $\gamma$ = 90° | $\gamma$ = 90° | $\gamma$ = 90° |
| Volume (Å$^3$) | 2014.0(2) | 2045.2(2) | 2080.4(8) | 2118.9(8) |
| $Z$ | 4 | 4 | 4 | 4 |
| Completeness (%) | 99.6 | 98.9 | 99.7 | 97.9 |
| $\mu$ (mm$^{-1}$) | 0.09 | 0.088 | 0.087 | 0.085 |
| $Dc$ (g/cm$^3$) | 1.320 | 1.300 | 1.278 | 1.255 |
| $R$(int) | 0.0628 | 0.087 | 0.0889 | 0.1105 |
| $R$(sigma) | 0.0885 | 0.1364 | 0.1187 | 0.1733 |
| Index ranges | $-8 \leq h \leq 9$ | $-8 \leq h \leq 9$ | $-9 \leq h \leq 9$ | $-8 \leq h \leq 9$ |
| | $-19 \leq h \leq 19$ | $-20 \leq h \leq 19$ | $-20 \leq h \leq 20$ | $-17 \leq h \leq 20$ |
| | $-16 \leq h \leq 24$ | $-16 \leq h \leq 24$ | $-17 \leq h \leq 26$ | $-25 \leq h \leq 24$ |
| GOF on $F^2$ | 1.008 | 1.008 | 1.018 | 0.945 |
| $R_1$ [$I > 2\sigma(I)$] | 0.0509 | 0.0701 | 0.0695 | 0.0759 |
| $wR_2$ [$I > 2\sigma(I)$] | 0.0915 | 0.1151 | 0.1128 | 0.1482 |

## Supplementary Methods.

**Detailed characterization of BDP1-6**. NMR spectra were recorded on a Bruker AVANCE III HD400. Chemical shifts (δ) were reported in ppm relative to $Si(CH_3)_4$ ($^1H$, $^{13}C$) and coupling constants (*J*) were given in Hz. Data for $^1H$ NMR are reported as follows: chemical shift (multiplicity, coupling constants where applicable, and number of hydrogens). Abbreviations are as follows: s (singlet), d (doublet), t (triplet), dd (doublet of doublet), m (multiplet). Mass spectra were obtained on a LCQ (ESI-MS, ThermoFinnigan) mass spectrometer.

**BDP1**: $^1H$ NMR (400 MHz, $CDCl_3$) δ/ppm = 7.49 (m, 3H), 7.38 (t, J = 7.3 Hz, 2H), 7.32 (m, 3H), 7.15 (m, 2H), 2.58 (s, 3H), 2.52 (s, 3H), 1.39 (s, 3H), 1.30 (s, 3H). $^{13}C$ NMR (100 MHz, $CDCl_3$) *δ*/ppm = 155.8, 154.2, 143.4, 142.1, 139.4, 129.2, 127.2, 121.5, 14.8, 14.6, 13.5, 12.8. ESI-MS: calcd, $[M]^+$ = 400.278, found, $[M]^+$ = 400.236.

**BDP4**: $^1H$ NMR (400 MHz, $CDCl_3$) δ/ppm = 8.61 (d, J = 5.1 Hz, 2H), 7.50 (m, 3H), 7.31 (m, 2H), 7.09 (d, J = 5.9 Hz, 2H), 6.05 (s, 1H), 2.59 (s, 3H), 2.54 (s, 3H), 1.40 (s, 3H), 1.31 (s, 3H). $^{13}C$ NMR (100 MHz, $CDCl_3$) *δ*/ppm = 157.9, 149.9, 142.5, 138.6, 135.0, 129.5, 129.4, 128.0, 125.2, 122.4, 15.0, 14.7, 13.3, 12.7. ESI-MS: calcd, $[M]^+$ = 401.266, found, $[M]^+$ = 401.269.

**BDP6**: $^1H$ NMR (400 MHz, $CDCl_3$) *δ*/ppm = 7.69 (m, 4H), 7.52 (dd, J = 4.8, 2.4 Hz, 2H), 7.34 (dd, J = 7.4, 2.0 Hz, 2H), 7.29 (m, 4H), 2.54 (s, 6H), 1.31 (s, 6H). $^{13}C$ NMR (100 MHz, $CDCl_3$) *δ*/ppm = 154.3, 143.4, 140.0, 138.8, 133.1, 131.7, 129.7, 127.9, 118.9, 111.2, 13.6, 13.0. ESI-MS: calcd, $[M]^+$ = 526.218, found, $[M]^+$ = 526.256.
